# Application of Ni-Ti Alloy connector for the treatment of comminuted coronal plane supracondylar-condylar femoral fractures: a retrospective review of 21 patients

**DOI:** 10.1186/1471-2474-14-355

**Published:** 2013-12-17

**Authors:** Yuntong Zhang, Xue Zhao, Yang Tang, Chuncai Zhang, Shuogui Xu, Yang Xie

**Affiliations:** 1 Department of orthopaedics, Changhai Hospital, Second Military Medical University, No.168, Changhai Street, Shanghai 200433, China

**Keywords:** Shape memory alloy, Treatment, Comminuted, Coronal plane, Femoral fracture

## Abstract

**Background:**

Our preliminary retrospective study assessed outcomes after the use of Ni-Ti arched shape-memory connector (ASC) combined with partially threaded cancellous screws (PTCS) to repair coronal plane supracondylar-condylar femoral fractures.

**Methods:**

Twenty-one patients (16 men and 5 women) with a mean age of 34.1 years (range, 28 to 44 years) with coronal plane supracondylar and condylar fractures of the distal femur were included in this study. Each patient underwent open reduction and internal fixation using the ASC and PTCS. Active functional exercises with restricted weight bearing were initiated the first postoperative day. A gradual increase in weight bearing status and range of motion was permitted and subjects progressed to full weight bearing by 8 weeks. Surgical time, blood loss, postoperative knee range of motion, American Knee Society Scores (KSS), and postoperative complications were assessed.

**Results:**

The mean surgical time was 75 mins (range, 45 to 100 mins) and average blood loss was 105 ml (range, 35 to 130 ml). Mean follow-up was 65 months (range, 22 to 90 months). No subjects demonstrated evidence of osteonecrosis or arthritis at the final follow-up. The mean KSS was excellent (≥85) in 8 subjects, good (70-84) in 11 subjects, and fair (60-69) in 2 subjects. The mean active range of motion of knee flexion at final follow-up was 100 degrees (range, 85 to 110 degrees).

**Conclusions:**

ASC combined with PTCS can serve as an effective means for managing comminuted femoral fractures that extend from the condyle to the supracondylar region. However, further prospective comparative studies and biomechanical analyses are needed to evaluate long-term outcomes using these materials.

## Background

Fractures of the isolated distal femoral condyle in the coronal plane were first described by Hoffa in 1904 [1]. This condition is rare and accounts for less than 1% of all femoral fractures, as it typically results only from high-velocity injuries [2,3]. Despite this low incidence, Nork and colleagues recently reported a 38% incidence of coronal plane supracondylar-intercondylar distal femoral fractures in adults [4]. This type of fracture has been reported to involve the lateral condyle more commonly [5], but fractures of the medial condyle have also been described [6]. Some authors have postulated that these fractures result from direct impact with the knee in a flexed position, while others have attributed the fracture to simultaneous vertical shear and twisting forces [5,7].

Operative treatment has been recommended for patients with condylar femoral fractures because surgical fixation provides stable restoration of the articular surface and facilitates early range of motion [5]. In recent years, several small series of cases were reported on outcomes after open reduction and internal fixation (ORIF) of Hoffa fractures [2-4,6,7]. Most of these cases were fixated using lag-screws for isolated Hoffa fractures, although the strength of this fixation may be insufficient for the comminuted fragments at the supracondylar distal femur region. Given that this is a rare type of fracture, the coronal fracture line that crosses the condyle to the supracondyle of distal femur has not been described by typical AO/OTA or Letenneur classification [8,9]. Accurate recognition of these injuries will assist with planning the surgical approach and determining the optimal implants for fixation. Furthermore, the use of Ni-Ti Alloy for the treatment of distal femur fractures has not been evaluated.

The goal of the study was to analyze the radiological and functional outcomes of patients after surgical treatment of comminuted coronal plane supracondylar-condylar femoral fracture using Ni-Ti arched shape-memory connector (ASC) and partially threaded cancellous screws (PTCS). We also reviewed the literature to highlight the importance of accurately detecting these fractures as well as describe the role of early and rigid internal fixation on patient outcomes.

## Methods

Twenty-one consecutive patients with comminuted coronal plane supracondylar and condylar fractures of distal femur underwent ORIF at our institution between August 1996 and August 2008. All procedures were approved by the Committee on Ethics of Biomedicine Research, Second Military Medical University. Written informed consent was obtained from the patients or from their relatives if the patients were incapable of consent for the publication of individual clinical details. All fractures were closed and there were no associated neurovascular injuries. Among the 21 patients (16 male and 5 female), the mean patient age was 34.1 years (range, 28 to 44 years). The mechanism of injury was a traffic accident for 14 patients and a fall from a substantial height for 7 patients. The 21 fractures were classified according to AO/OTA guidelines [8] as type 33-B3. However, in all cases the fracture line crossed from the condyle to the supracondylar region and the supracondyle fracture was comminuted into more than three fragments. Eight fractures involved the lateral condyle and thirteen involved the medial condyle. Five patients had fractures in the right knee 16 patients had fractures of the left. Patellar fractures occurred in 5 patients and 3 patients had fractures in other extremities. Medial collateral ligament (MCL) injury occurred in 5 patients and 2 patients had medial meniscus tears confirmed by MRI. Patient characteristics are shown in Table 1.

**Table 1:** Summary of the patient profiles and subsequent management with outcomes

**No.**	**Age/sex**	**Mechanism of injury**	**Associated injuries**	**Modality of internal fixation for distal fumoral fractures**	**Knee ROM (in degrees)**	**Knee Society scores**
1	31/F	fall	/	ASC×3&PTCS×2(6.5 mm)	0-100	83/good
2	38/M	Traffic accident	Tibial plateau fracture/medial meniscus tears	ASC×3&PTCS×1(6.5 mm)	5-100	79/good
3	39/M	Traffic accident	/	ASC×2&PTCS×2(4.5 mm)	0-120	88/excelent
4	32/M	fall	Calcaneus and patellar fracture / MCL injury	ASC×3&PTCS×2(6.5 mm)	0-105	90/excelent
5	34/M	fall	/	ASC×2&PTCS×1(4.5 mm)	5-95	84/good
6	35/M	fall	/	ASC×2&PTCS×1(6.5 mm)	0-105	82/good
7	44/M	Traffic accident	Tibial plateau fracture/MCL injury/ medial meniscus tears	ASC×3&PTCS×2(4.5 mm)	0-115	92/excelent
8	28/F	Traffic accident	Patellar fracture	ASC×2&PTCS×2(6.5 mm)	0-95	88/excelent
9	22/M	Traffic accident	MCL injury	ASC×2&PTCS×1(6.5 mm)	0-110	93/excelent
10	33/M	Traffic accident	medial meniscus tears/MCL injury	ASC×3&PTCS×2(4.5 mm)	0-90	65/fair
11	39/M	fall	/	ASC×2&PTCS×2(4.5 mm)	0-95	90/excelent
12	32/F	Traffic accident	Patellar fracture	ASC×3&PTCS×2(6.5 mm)	0-90	80/good
13	30/M	Traffic accident	/	ASC×2&PTCS×1(6.5 mm)	0-85	68/fair
14	43/F	Traffic accident	/	ASC×3&PTCS×2(4.5 mm)	5-115	90/excelent
15	29/M	Traffic accident	/	ASC×2&PTCS×1(6.5 mm)	0-105	84/good
16	30/M	Traffic accident	MCL injury	ASC×3&PTCS×2(4.5 mm)	5-95	82/good
17	38/M	Traffic accident	Patellar fracture	ASC×2&PTCS×1(6.5 mm)	5-110	82/good
18	34/M	fall	/	ASC×3&PTCS×2(6.5 mm)	5-100	78/good
19	38/F	fall	Patellar fracture	ASC×3&PTCS×2(6.5 mm)	0-100	84/good
20	34/M	Traffic accident	/	ASC×3&PTCS×2(6.5 mm)	5-115	92/excelent
21	33/M	Traffic accident	/	ASC×3&PTCS×2(4.5 mm)	5-110	82/good

*ROM*: range of motion, *ASC*: Ni-Ti arched shape-memory connector, *PTCS*: partially threaded cancellous screws.

### Structure and working principle of the ASC device

**Figure 1:**
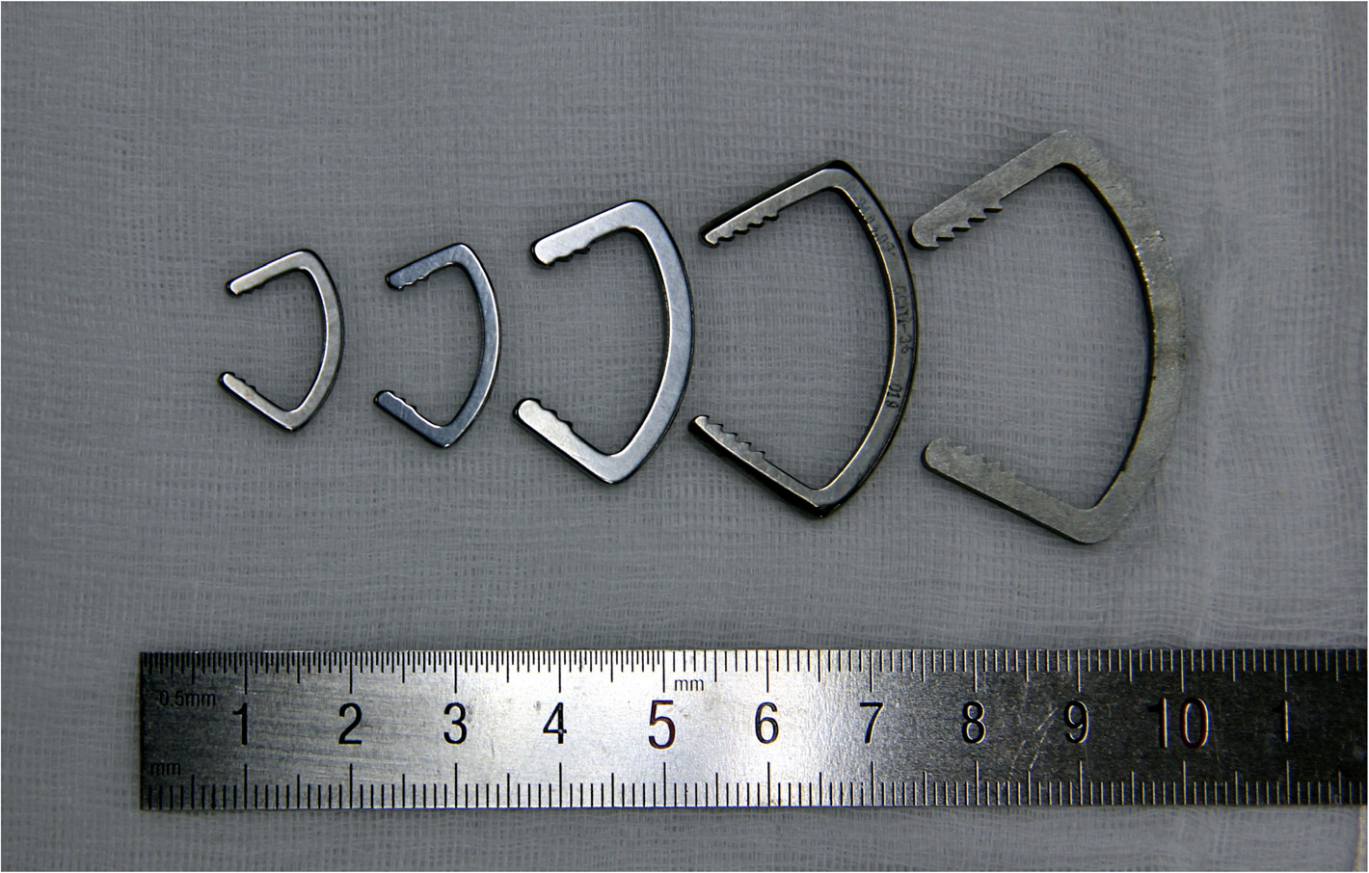
Ni-Ti arched shape-memory connectors.

**Figure 2:**
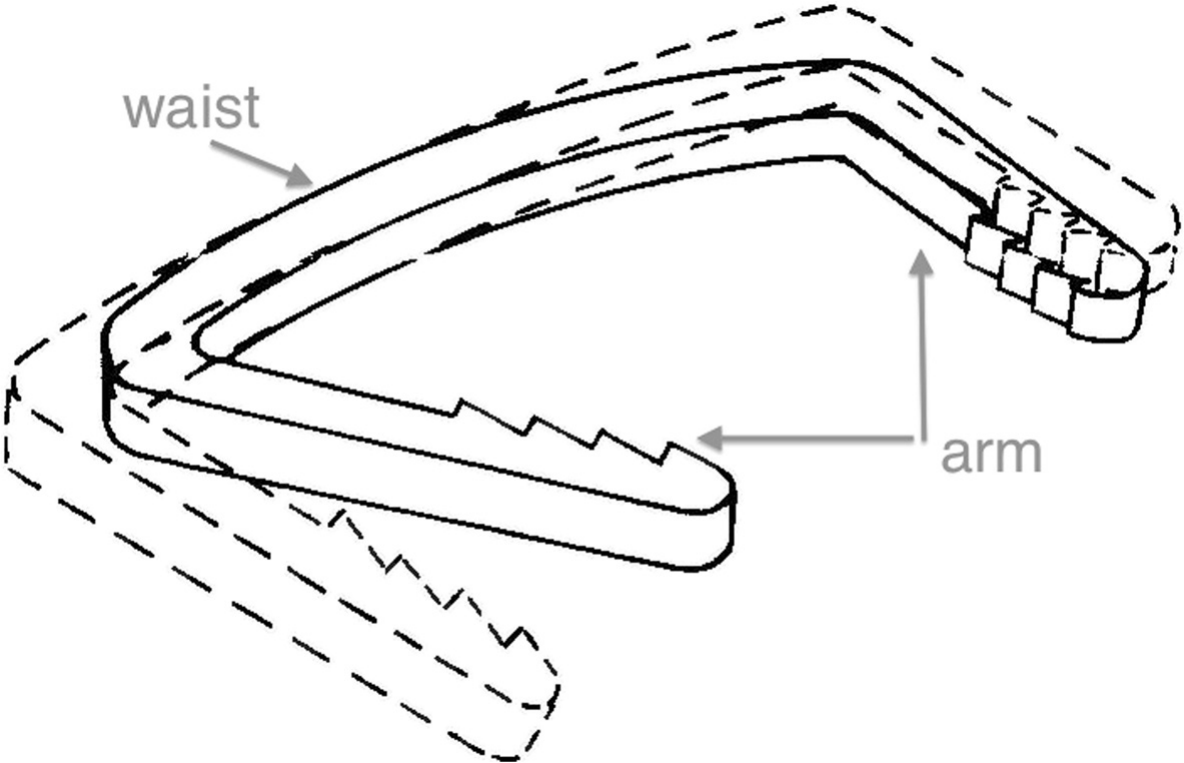
**The device consists of compression arms that are connected to the waist**.

Ni-Ti shape-memory alloys possess certain novel properties, particularly they have the ability to return to their initialized shape via a simple change of temperature. This unique property allows a variety of applications in implantology. Although many of these products are still in the research and development stage, some have been incorporated into industrial products. We designed a Ni-Ti shape-memory alloy device that has been applied to treating upper and lower extremity fractures, including hip, digit, and scaphoid fractures [10-14]. Given the rounded shape of the device, we have identified this particular device as the arched shape-memory connector (ASC, Figure 1). The ASC device (Huzhou Swan Biological Memory Medical Devices Co., Ltd., Zhejiang, China.) was manufactured with 2 mm thick Ti-Ni shape-memory alloy (with Ni content of 50-53%). The device (Figure 2), processed with one-way heat treatment and a reversion temperature of 33 ± 2°C, consisted of compression arms that were merged at the device waist. Before implantation, the ASC was placed in 0-4°C ice water for cooling because it is malleable at lower temperatures (martensite). After fixation, 40-50°C water was used to warm the device to activate its memory mechanical functions (austenite).

### Surgical procedures

**Figure 3:**
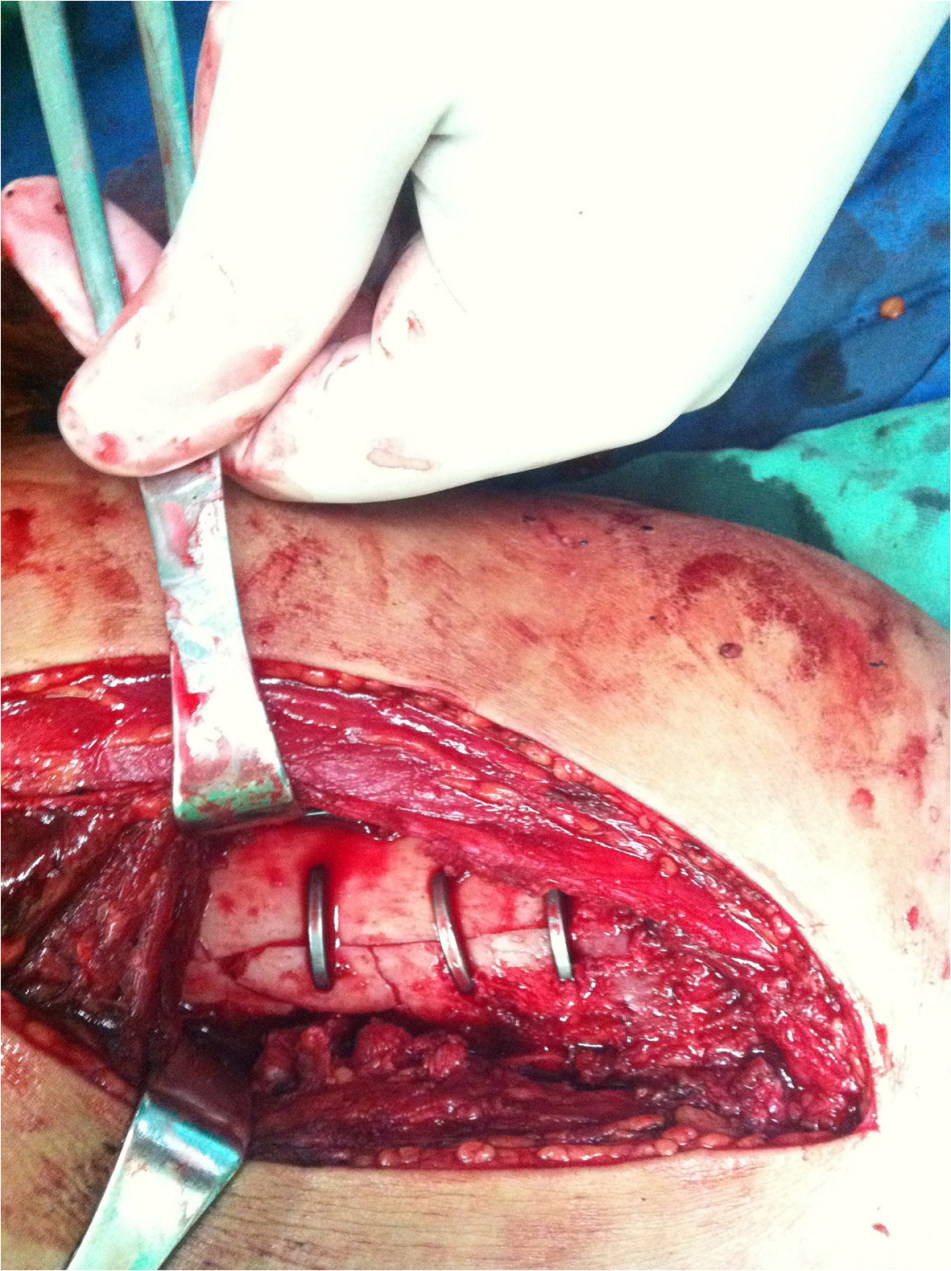
Intraoperative picture of ASC for fixation of comminuted supercondylar fractures.

The surgical approach was based on associated injuries. A midline incision with a medial/lateral parapatellar arthrotomy or direct lateral approach was used for the ORIF. In all cases, the fracture was anatomically reduced in knee flexion. 4.5 mm or 6.5 mm partially threaded cancellous screws (PTCS) with or without washers were used for AP fixation. The ASC was immersed in ice water, and flattened as shown in the dotted line of Figure 1. Then, two holes were drilled on either side of the fracture site at the femoral supracondylar region for attachment of the arms of the ASC. After being fixated into bone, the ASC was heated with 40-50°C warm water and the arm and waist of ASC reverted to its original shape. Because the two arms of ASC were fixated around the fracture, the reverting force produced compressive stress at the fracture line (Figure 3). Associated fractures and tissue injury were treated as appropriate. Active exercises with restricted weight bearing were started on the first postoperative day. A gradual increase in weight bearing status and range of motion was then permitted and all patients progressed to full weight bearing by 8 weeks.

## Results

The mean surgical time was 75 mins (range, 45 to 100 mins) and average blood loss was 105 ml (range, 35 to 130 ml). Direct lateral approach was used 17 patients, and medial parapatellar arthrotomy was used in 4 patients. All wounds healed uneventfully without deep or superficial skin infection. 6.5 mm AP screws were used in 13 patients and 4.5 mm screws were used in the remaining 8 patients. Articular reductions were classified as anatomical, acceptable (<2 mm step) or poor (>2 mm) based on the immediate postoperative radiographs. All 21 fractures were anatomically reduced. AP and lateral X-rays were taken at each follow up visit. The average follow-up time was 65 (range, 22 to 90) months. Patients ambulated with full weight bearing an average of 1.6 (range, 1.2 to 2.1) months after surgery. Union was defined using a combination of clinical and radiographic criteria. To be classified as union of the fracture, the patient must have been able to bear weight on the limb without pain and also have obliteration of the fracture line or evidence of bridging trabeculae across the fracture line.. The mean time of bone union was 7 weeks (range, 6 to 9 weeks). No comminuted osteoporotic fracture, implant failure, secondary loss of reduction, delayed union or nonunion was observed in any patient. At final follow-up, KSS was excellent (≥85) in 8 patients, good (70-84) in 11 patients, and fair (60-69) in 2 patients. There was no evidence of osteonecrosis or traumatic arthritis in any patient at any follow-up visit. The mean active range of motion of knee flexion at final follow-up was 100 degrees (range, 85 to 120 degrees). No other intraoperative or postoperative complication occurred. None of the patients required hardware removal. Patient outcomes are summarized in Table 1.

### Representative case

**Figure 4:**
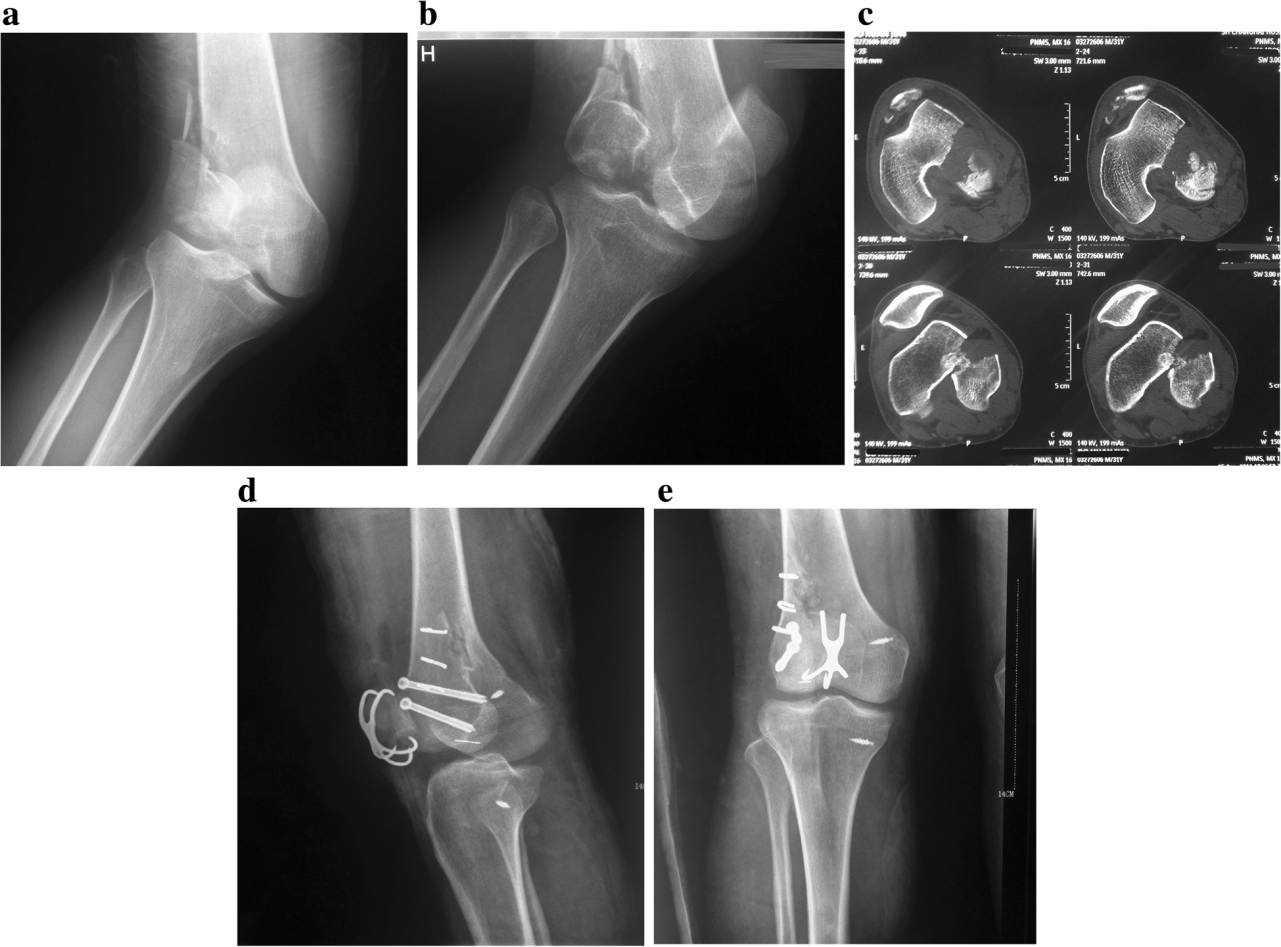
**Representative case. (a,b)** a 32-year-old male patient was presented with comminuted supracondylar-condylar fracture of the right distal femur, associated with patellar fracture and medial collateral ligament injury. **(c)** The CT shows coronal plane fracture of supracondylar-condylar femur. **(d,e)** Post-operation radiographs with 30 months follow-up indicate the fracture has healed well. No evidence of osteonecrosis or development of traumatic arthritis can be observed.

A 32-year-old male patient presented with comminuted supracondylar-condylar fracture of the right distal femur. He had an associated patellar fracture and medial collateral ligament injury (Figure 4-a,b). The CT revealed a coronal plane fracture of supracondylar-condylar femur (Figure 4-c). Post-operative radiographs showed the supracondylar-condylar fracture was anatomically reduced and fixed with three ASCs and two 6.5 mm AP screws, (Figure 4-d,e). Post-operative radiographs at the 30 month follow-up indicated that the fracture was well healed well. No evidence of osteonecrosis or development of traumatic arthritis was observed (Figure 4).

## Discussion

A comminuted condylar fracture of the distal femur, associated with a coronal split of both the condyle and supracondyle is a challenge to diagnosis and treat. Most cases present with an obviously swollen knee joint, haemarthrosis and pain; however, the diagnosis is best made by radiographs. Displaced condylar fragments can be seen on lateral radiographs, but minimally displaced or even undisplaced fractures in the coronal plane can be easily missed. Computerized Tomography (CT) scans should be performed cases in which these fractures are suspected based on clinical presentation and mechanism of injury.

Coronal plane supracondylar-condylar femoral fractures are intra-articular and inherently unstable. Most authors conclude that conservative treatment, including closed reduction and cast immobilization or prolonged traction, places the patient at risk for displacement of fracture fragments and future nonunion [15-18]. A long period of immobility time can result in joint stiffness, poor joint physical function and subsequent osteoarthritis [19]. Based on these potential poor prognostic factors, these fractures should be treated via open or arthroscopic reduction and internal fixation. A midline incision with a medial/lateral parapatellar arthrotomy is the most common approach reported. Direct lateral approach with or without osteotomy of the Gerdy tubercle and posterior-based approaches have also been described [20,21]. Holmes et al [18] proposed a standardized surgical approach to the fracture and rigid fixation with optimally positioned lag screws placed perpendicularly to the fracture plane. They reported that an ipsilateral parapatellar approach provided optimal visualization of the fracture and the articular surface as necessary to achieve a perfect anatomic reduction and rigid internal fixation. This technique may benefit early and unrestricted range of motion exercises, which lowers the risk of knee joint ankylosis. We feel that either the medial parapatellar arthrotomy or direct lateral approach is appropriate, as both provide good visualization of the fracture and the articular surface. Additionally, these approaches are familiar to most orthopaedic surgeons who perform total knee replacements.

There are relatively few recommendations in the literature for fixation of coronal fractures of the posterior femoral condyles. Benirschke and Swiontkowski suggested the use of 3.5 mm cortical lag screws [22]. Liebergall et al recommended use of 6.5 mm cancellous screws [23]. Mize suggested that K-wires and absorbable pins are not strong enough and recommended use of 4.0 mm cancellous or similar screws [24]. Jarit et al. reported that PA screws showed less displacement than AP screws when subjected to vertical loads [25]. However, the PA screws need to be countersunk, which can result in cartilage damage. This was addressed in the current study using headless screws [26], although these screws have the disadvantage of small size and length. Most of reports of these fractures include simple fractures with two or three big fragments. For comminuted fractures of the supracondylar-condylar region of the femur, using screws alone cannot provide anatomical reduction of the joint with stable internal fixation that permits early range of motion.

Recently, the Less Invasive Stabilization System has been developed to manage distal femur fractures. This system uses multiple, fixed-angle, distal locked screws and can be best thought of as a submuscular “internal” fixator [27]. Multifragmentary fractures of the metaphysis are reduced by indirect means and stabilized by plates, which act as bridge plates; however, the metaphyseal part of the fracture is never visualized [28]. Using the principles of minimally invasive plate osteosynthesis the plate is slid underneath the muscle and external to the periosteum [29]. This form of closed reduction without additional traumatization of the diaphyseal area leads to improved fracture healing and improved local resistance to infection [30]. However, surgical exposure of the site using this method is limited. This carries with it an inherent risk of postoperative malrotation and malalignment, especially in intra-articular fractures [30,31].

The current trend in the treatment of periarticular fractures is the use of small fragment implants that are lower profile and necessitate less periosteal and soft tissue disruption. To avoid the need for a distal femoral locking plate, we implemented ASCs as adjuvant to partially threaded cancellous screws internal fixation for comminuted coronal plane supracondylar-condylar femoral fractures.

Ni-Ti shape-memory alloy, as a functional metal material, possesses excellent properties of wear and corrosion resistance and good biocompatibility, as well as shape-memory effect. In 1990, the FDA (USA) approved this alloy for medical use. Medical applications of this material expanded and Ni-Ti shape-memory alloy was widely regarded as a “valuable biological memory material” [32]. In the past decade, shape-memory alloys have been gradually applied to various clinical fields of practice including stomatology and orthopedics [32,33]. ASC has been shown to be an effective means for the treatment of humeral shaft nonunions, scaphoid waist nonunions and acetabular fractures [10-14]. Despite the widespread use for a variety of fractures, no clinical studies on its use in distal femoral fractures have been reported.

Compared with other methods, ASC with associated PTCS is advantageous for the following reasons: 1) Screws fixing posterior femoral condyle fractures are usually placed through articular cartilage. The ASC can be placed at the lateral side to minimize the damage to the articular cartilage, which may reduce the development of post-traumatic arthritis (Figure 4-e). 2) As a rigid and reliable fixation method, traditional medial/lateral femoral blade plates have been used for comminuted fractures of the distal femur. However, the use of these plates requires wide exposure and greater disruption of the periosteum and soft-tissue. Minimally invasive techniques such as the Less Invasive Stabilization System may eliminate the need for a wide area of surgical exposure; however, open reduction and using the ASC for coronal supracondylar fractures may be superior considering the quality of reduction, short operation time, need for minimal soft tissue dissection, and low risk of vascular injury and blood loss. 3) The fixation material should have a modulus equivalent to that of bone. Current implant materials have higher stiffness than bone, which prevents the needed stress being transferred to adjacent bone. This biomechanical alteration can lead to excessive resorption and subsequent implant loosening through the “stress shielding effect” [34]. ASC is a functional fixator with excellent combination of high strength and low modulus that is more akin to that of bone [35]. In addition, due to the inherent property of shape-memory alloy, the compression arms in the device provide evenly distributed compression forces. These forces provide continuous axial compression on the fractured ends, which can be transmitted across the fracture to ensure the stable biomechanical environment that allows smooth healing of the fracture line. In our patients, fewer screws are required and full healing was achieved in 7 weeks, similar to other fixation methods [4,5,7].

Although this preliminary study revealed the feasibility of using ASC for distal femoral fixation, several limitations exist. This study was retrospective, patients were not randomized to evaluate comparative effectiveness and our sample size was too small to perform statistical analysis. However this is the first report to describe the use of ASC in comminuted coronal plane supracondylar-condylar femoral fractures.

## Conclusions

ASC combined with PTCS can serve as an effective means for managing comminuted coronal plane supracondylar-condylar femoral fractures. However, further prospective comparative studies and biomechanical analyses are needed to evaluate long-term outcomes using these materials.

## Competing interests

The authors declare that they have no conflicting interests.

## Authors’ contributions

YZ, YX and SX contributed to the study concepts, literature research and manuscript preparation. CZ and YX are the guarantors of integrity of the entire study and participated in the operation and manuscript review. XZ, YT and SX participated in literature research, data acquisition and data analysis. All authors read and approved the final manuscript.

## Pre-publication history

The pre-publication history for this paper can be accessed here:

http://www.biomedcentral.com/1471-2474/14/355/prepub
